# Simultaneous Detection and Genotyping of Hydatid Cysts in Slaughtered Livestock via a Direct PCR Approach

**Published:** 2019

**Authors:** Jiafei ZHAN, Ning WANG, Ruiqi HUA, Nengxing SHEN, Yue XIE, Xiaobin GU, Weiming LAI, Xuerong PENG, Guangyou YANG

**Affiliations:** 1. Department of Parasitology, College of Veterinary Medicine, Sichuan Agricultural University, Chengdu 611130, China; 2. Sichuan Academy of Chinese Medicine Sciences, Chengdu 610041, China; 3. College of Bioengineering, Sichuan University of Science & Engineering, Yibin 644000, China; 4. College of Science, Sichuan Agricultural University, Ya’an 625014, China

## Dear Editor-in-Chief

Cystic echinococcosis (CE) is a parasitic zoonosis caused by the larval stage (hydatid cysts) of *Echinococcus granulosus sensu lato (*s.l.), resulting in large economic losses to livestock husbandry (1). A high degree of genetic diversity between *E. granulosus* strains (G1, G3, G4–G8, and G10) is one of main features of this parasite, and identifying its genotype would be advantageous for prevention and control of the disease (2).

Macroscopic diagnosis at necropsy alone would resulted in a 15.4% error due to the variability of cystic morphology (3). If the cysts are small (especially below 0.2 cm), develop incomplete as well as present on uncommon parasitic sites, it would more difficult to accurately identify and distinguish them from other metacestodes of the family Taeniidae, or some cyst-like lesions (unspecific granulomas, tumours and abscesses).

Compared with visual inspection, molecular techniques, especially PCR assays using mitochondrial markers (e.g., COX1 and ND1), are being increasingly applied to CE clinical detection due to the accuracy and specificity (4). The PCR assay usually includes DNA extraction, PCR amplification and then amplification product sequencing, of which the first two steps are time consuming. To overcome this problem, a direct PCR assay has been emerged from conventional PCR and applied in parasitic species including helminths, protozoans and insect vectors (5).

Herein, we specifically developed a direct PCR assay for detection and genotyping of hydatid cysts in slaughtered livestock by amplifying the complete mitochondrial NADH dehydrogenase subunit 6 gene (ND6, 457bp) of *E. granulosus* s.l.

In this study, the lysate of washed hydatid cysts, treated by an Animal Tissue Direct PCR Kit (Foregene, Chengdu, China) within 0.5 h, could be directly used as template in our PCR protocol. Then, the forward (5′-TTTCGTGCTGTAGATGGT-3′) and reverse (5′-CACAGATTTCAAAGGGTT-3′) primers designed based on the conserved regions of eight genotypes of *E. granulosus* s.l., were used to amplify the complete ND6 gene (558 bp). PCR reaction volume contained 10 μL 2×PCR Easy Mix (containing D-Taq DNA polymerase), 0.5 μL of each primer (20 mM), 4.0 μL of lysate and 5 μL of double-distilled water (dd H_2_O). Thermal cycling proceeded within 1 h, and conditions included an initial denaturation at 95 °C for 3 min, followed by 30 cycles of denaturation at 95 °C for 10 s, annealing at 58°C for 20 s, and extension at 72 °C for 20 s, then a final extension at 72 °C for 5 min. All testing were performed and analysed in triplicate.

**Fig. 1:**
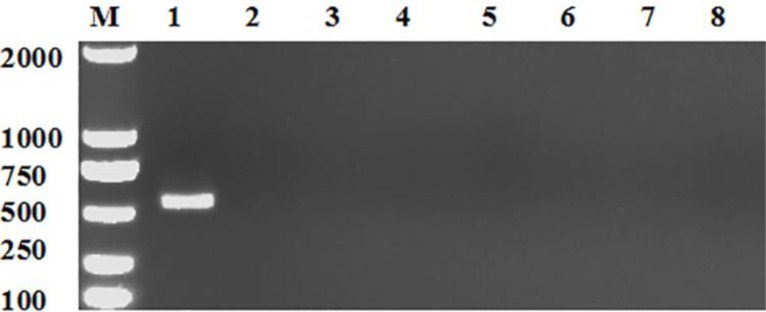
Specificity of the direct PCR assay for detection of hydatid cysts M, DL2000 DNA markers; Lane 1, Hydatid cysts; Lane 2, *Echinococcus alveolaris*; Lane 3, *Cysticercus tenuicollis*; Lane 4, *Coenurus cerebralis*; Lane 5, *Cysticercus bovis*; Lane 6, *Cysticercus cellulosae*; Lane 7, *Cysticercus viscerotropica*; Lane 8, Negative control

**Fig. 2:**
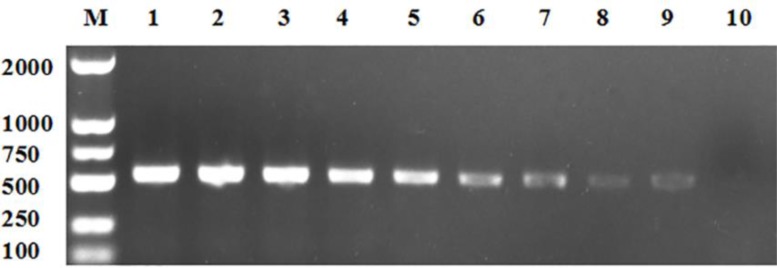
Sensitivity of the direct PCR assay for detection of hydatid cysts M: DL2000 DNA markers; Lane 1: 500 ng DNA; Lane 2: 50 ng DNA; Lane 3: 10 ng DNA; Lane 4: 2 ng DNA; Lane 5: 400 pg DNA; Lane 6: 80 pg DNA; Lane 7: 16 pg DNA; Lane 8: 8 pg DNA; Lane 9: 4 pg DNA; Lane 10: 2 pg DNA

The results showed that DNA of lysate of hydatid cysts could be used for this direct PCR amplification, and the amplified sequences shared a 100% identity with published ND6 gene sequence of *E. granulosus* (Accession Nos. KJ559023.1). No cross-reactivities were found between hydatid cysts and six other metacestodes commonly found in China including *E. alveolaris, Cysticercus tenuicollis, Coenurus cerebralis, C. bovis, C. cellulosa* and *C. viscerotropica*, indicating a high species specificity of this method (Fig. 1). The sensitivity analysis using serially diluted DNA with concentrations ranging from 500 ng to 2 pg revealed that DNA yielded specific bands with a detection sensitivity as low as 4 pg of DNA, indicating a high sensitivity (Fig. 2).

In addition, out of 72 ambiguous hydatid cysts from the slaughter houses of Qinghai and Xinjiang provinces, 94.4% (68/72) samples were positive by the direct PCR. Moreover, the sequence data of ND6 genes showed that 65 cysts belonged to G1 genotype and three cysts belonged to G3 genotype, which were the same as the genotyping results of the COX1 gene (not shown), suggesting the ND6 gene can be also a good genetic marker for the genotyping of *E. granulosus*.

In conclusion, the direct PCR assay developed in this study displayed a high specificity, sensitivity and effectiveness. Moreover, a low cost with ∼$0.58 per should make it an ideal tool for large-scale CE clinical detection in future.

## References

[1] Wen H Lucine V Tuxun T Echinococcosis: Advances in the 21st Century. Clin Microbiol Rev. 2019; 32(2):e00075–18. 30760475 10.1128/CMR.00075-18 PMC6431127

[2] Lautimäe T Kinkar L Moks E Molecular phylogeny based on six nuclear genes suggests that *Echinococcus granulosus* sensu lato genotypes G6/G7 and G8/G10 can be regarded as two distinct species. Parasitology. 2018; 145(14): 1929–1937. 29781421 10.1017/S0031182018000719

[3] Barnes TS Deplazes P Gottstein B Challenges for diagnosis and control of cystic hydatid disease. Acta Trop. 2012; 123(1):1–7. 22410539 10.1016/j.actatropica.2012.02.066

[4] Bowles J Blair D Mcmanus DP Genetic variants within the genus *Echinococcus* identified by mitochondrial DNA sequencing. Mol Biochem Parasitol. 1992; 54(2): 165–73. 1435857 10.1016/0166-6851(92)90109-w

[5] Wang N Wang Y Ye Q Development of a direct PCR assay to detect *Taenia multiceps* eggs isolated from dog feces. Vet Parasitol. 2018; 251:7–11. 29426480 10.1016/j.vetpar.2017.12.017

